# Association of serum brain‐derived neurotrophic factor level and early response to antipsychotic drug in first‐episode patients with schizophrenia

**DOI:** 10.1002/mpr.1982

**Published:** 2023-07-23

**Authors:** Tong Zhao, SuFang Tang, XiaoLei Gao, Juan Li, Ran Hao, HaiZhi Chen, GuangBiao Huang

**Affiliations:** ^1^ Department of Psychiatry QuZhou Third Municipal Hospital QuZhou China; ^2^ Department of Psychiatry Huzhou Third Municipal Hospital The Affiliated Hospital of Huzhou University Huzhou China; ^3^ School of Nursing Xinxiang Medical University Xinxiang Henan China; ^4^ Department of Psychiatry The Second Affiliated Hospital of Xinxiang Medical University Xinxiang Henan China; ^5^ The First Affiliated Hospital of Xinxiang Medical University Xinxiang Henan China

**Keywords:** antipsychotic drugs, brain‐derived neurotrophic factor, psychotic symptoms, risperidone, schizophrenia

## Abstract

**Objectives:**

To investigate the role of Brain derived neurotrophic factor (BDNF) in the psychotic symptoms in first‐episode patients with schizophrenia and whether BDNF levels were associated with the improvement of psychotic symptoms after risperidone treatment.

**Methods:**

89 schizophrenia patients and 90 healthy controls were recruited, the schizophrenia patients were assigned into early response or early non‐response groups at 2 weeks based on improvement in the positive and negative symptoms scale (PANSS) total score. All patients were treated with risperidone for 2 weeks, their serum BDNF levels were compared at baseline and after 2 weeks treatment.

**Results:**

We found that patients had lower BDNF levels, compared to controls at baseline. After 2 weeks of treatment of risperidone, BDNF levels were significantly increased and psychotic symptoms were decreased in early response group. Correlation analysis showed that the change of BDNF levels after treatment was correlated with the change of PANSS total score. Further regression analysis showed that the change in BDNF levels was an independent predictor for the improvement in psychotic symptoms.

**Conclusions:**

Our findings reveal that the level of BDNF was lower in first‐episode schizophrenic patients, moreover, the changes in serum BDNF levels may have a predictive effect on the early improvement in psychotic symptoms in the first 2 weeks.

## 1 INTRODUCTION

How long an antipsychotic should be trialed before being viewed as ineffective is a key unanswered question in clinical trials for patients with schizophrenia. In many studies, most of the symptomatic improvement seen in response to atypical antipsychotic therapy occurs in the first 1–2 weeks, with effects seen in some patients in as early as 24 h (Agid et al., 2003; Svanum et al., 2011). Investigations indicated that response to antipsychotic treatments begins in the ﬁrst weeks of treatment with the largest effect in reducing symptoms in the ﬁrst 2 weeks (Agid et al., 2003; Murray et al., 2017). Early nonresponse was operationally deﬁned as “<20% improvement on positive and negative symptoms scale (PANSS) or brief psychiatric rating scale (BPRS) total score at 2 weeks” (Kinon et al., 2010). Compared with patients who lack at least minimal symptom improvement following 2 weeks of treatment (‘early non‐responders’), early responders were previously found to have greater improvement in symptoms and functioning, a higher symptom remission rate (Kinon et al., 2010; Svanum et al., 2011) and lower health care costs (Svanum et al., 2011). Early response to antipsychotic drug therapy may be a clinical marker of subsequent response in the treatment of schizophrenia (Stauffer et al., 2011), It also help clinicians optimize the initial choice of treatment for a given patient. Thus, it is important when possible to identify patients as early responders or early non‐responders, and consider alternative treatment options for patients who are less likely to respond. However, so far, no distinct laboratory test or brain scan or any other bio‐marker is available to distinguish early responders or early non‐responders to antipsychotic drug in first‐episode patients with schizophrenia.

Brain derived neurotrophic factor (BDNF) is thought to be involved in the pathogenesis of schizophrenia (Zhang et al., 2013). It is well‐known that BDNF can cross the blood‐brain barrier (Pan et al., 1998), In some case control studies, peripheral blood and cerebro spinal fluid (CSF) levels of the BDNF protein were also decreased in patients with schizophrenia (Cakici et al., 2020). Some reports have shown that serum BDNF levels decreased in the treated schizophrenia patients (Pinto et al., 2017; Rodrigues et al., 2018; Bora, 2019), others failed to identify any significant differences between patients and healthy individuals (Chang et al., 2006). It has been revealed that serum BDNF levels in the patients treated with clozapine are higher than those treated with risperidone or typical antipsychotics (Dong et al., 2021), However, it has been shown that serum levels of BDNF of patients with schizophrenia do not raise after antipsychotic treatment (Pirildar et al., 2004). Nevertheless, Rizos et al. have stated that serum BDNF levels were significantly increased in the patients treated with olanzapine compared to those treated with haloperidol, risperidone, and amisulpride (Rizos et al., 2010). Previous findings showing that BDNF may act as a candidate marker of schizophrenia, and its serum changes have been linked with the response to treatment with antipsychotics (Angelucci et al., 2000; Han et al., 2021).

Despite the attributes of the schizophrenia, few studies have been undertaken to investigate whether there is a difference in serum BDNF level between early responders and early non‐responders to antipsychotic drug in first‐episode patients with schizophrenia. We hypothesized that the quantity of positive and negative syndrome in schizophrenia undergoing antipsychotic treatment may differ between early responders and early non‐responders individuals. To test this hypothesis, we assessed positive and negative syndrome scale parameters of early responders and non‐responders schizophrenia. Furthermore, to explore the underlying molecular mechanisms for early responses after antipsychotic treatment, we assessed the effects of antipsychotic treatment in early responders and non‐responders patients on levels of BDNF, which may be one of several mechanism of early response and had been connected with the reaction to treatment with antipsychotics.

## 2 METHODS

### 2.1 Participants

This study recruited first‐episode male adult patients of schizophrenia, who were not received neuroleptic treatment before this investigation. They have to meet the diagnostic criteria for schizophrenia according to the Diagnostic and Statistical Manual of Mental Disorders, Fourth Edition (DSM‐IV). All subjects provided written informed consent to participate in the study, which was approved by the Ethics Committee of Huzhou Third Municipal Hospital, China [Date of the permission by Ethics Committees: May 29, 2014; permission number: (2014)伦审第(12)号] and was conducted in accordance with the latest version of the Declaration of Helsinki.

The inclusion criteria for this study were: (1) aged 16–40 years, whose onset of psychotic symptoms first occurred before the age of 36, (2) no major systemic illnesses based on physical examinations and laboratory test results, and (3) baseline PANSS total score ≥60 (Chang et al., 2006). The exclusion criteria were as follows: (1) prior antipsychotic drug treatment of more than 16 cumulative weeks in the patient's lifetime, or take the medicine for more than 3 days at a time (Because of a disease other than schizophrenia); (2) recent use of injectable depot neuroleptics; (3) any prior treatment with clozapine; (4) pregnant or breastfeeding; (5) serious unstable medical condition; (6) DSM‐IV substance dependence (except caffeine and nicotine) within the past month; (7) patient judged to be suicidal or too seriously ill to be included; (8) pre‐morbid IQ ≤70; (9) or past history of any DSM‐IV psychotic disorder with recovery.

### 2.2 Clinical evaluation and grouping

PANSS rating scale was used to evaluate the changes of psychiatric symptoms in each time point. Prior to the present study, all participating psychiatrists had received adequate training through the manual and they had clinical experience in the PANSS rating before the study. At each time point if the scores of PANSS showed the patient's symptoms had worsened, the dosage would be adjusted based on the clinical judgment of in‐charged senior psychiatrist.

After screening, eligible patients were assigned to receive a risperidone for 2 weeks. At the end of this study, the dosage of risperidone ranging from 3 mg/d to 5 mg/d. If needed, Lorazepam was used to treat the insomnia syndrome or drug‐induced akathisia with a dosage of 1 mg/d and Trihexyphenidyl was used to treat the extrapyramidal syndrome with a dosage of 4 mg/d.

Patients were assigned into early response (ER) or early non‐response (ENR) groups at 2 weeks based on improvement in the PANSS total score. Early responders showed ≥20% improvement in PANSS total score from baseline. Early non‐responders showed <20% improvement in PANSS total score from baseline.

Healthy controls (HC) were recruited from the community. A psychiatrist excluded potential individuals with past/current mental disorders by using DSM‐IV. We also excluded HCs who received antipsychotics. The patients were matched well to the healthy control subjects regarding age (*p* = 0.2041).

### 2.3 Sample collection and measurement of biochemical indicators

Following an overnight fast, 10 mL of peripheral blood from all participants were collected in the morning (06:00 am to 9:00 am). We separated the plasma through 15 min high speed (3000 g) cryogenic (4°C) centrifugation, then stored at −80°C before used. The ELISA kits (R&D Systems, Minneapolis, Minnesota, USA) were used to measure the serum BDNF levels.

### 2.4 Statistical analyses

SPSS 19.0 for Windows was used to analyze the collected data. Data were generally reported as the mean ± SD. Independent sample *t*‐test were performed for the comparisons of some demographic and clinical variables between early response, early non‐response and health control subjects. The significance of differences observed among multiple groups was evaluated with one‐way ANOVA. Relationships between BDNF level and PANSS total scores were evaluated using Pearson correlations. A difference was considered significant at two tailed *p* < 0.05.

## 3 RESULTS

### 3.1 Baseline and improvement of BDNF levels comparison between ER patients, ENR patients and HCs

Table 1 shows the age and BDNF levels of all participants. These was no significant between ER group, ENR group and HC group in age (F(2,176) = 1.604, *p* = 2.041). Contrast with HC group, the patients in both ER and ENR group had lower baseline BDNF levels (*t* = 7.476, *p* < 0.001; *t* = 8.902, *p* < 0.001). Furthermore, no significant difference was seen between ER and ENR group in baseline BDNF levels (*t* = 1.87, *p* = 0.065).

**TABLE 1:** Brain derived neurotrophic factor (BDNF) level at baseline and after 2 weeks of treatment.

Variable	HC(*n* = 90) mean ± S.D.	ER (*n* = 41) mean ± S.D.	ENR (*n* = 48) mean ± S.D.	Analysis 1		Analysis 2		Analysis 3		Analysis 4		Analysis 5	
				*t*	*p*	*t*	*p*	*t*	*p*	*t*	*p*	*t*	*p*
Age in years	29.79 ± 5.98	30.23 ± 6.89	29.35 ± 6.56	−1.149	0.253	−0.475	0.635						
BDNF level Baseline	1552.04 ± 275.04	1188.50 ± 182.50	1095.51 ± 264.45	7.476	<0.001**	8.902	<0.001**	1.87	0.065				
BDNF level 2 weeks later		1335.99 ± 255.70	1169.41 ± 321.44	4.073	<0.001**	6.875	<0.001**	2.632	0.010*	−2.969	0.004**	−1.204	0.232

*Note*: Analysis 1: ER group versus HC group; Analysis 2 ENR group versus HC group; Analysis 3 ER group versus ENR group; Analysis 4: ER group, 2 weeks later versus baseline; Analysis 5: ENR group, 2 weeks later versus baseline. The unit of measurement for BDNF:µg/L.

Abbreviations: ENR, early non‐response; ER, early response; HC, healthy control.

*p < 0.05, **p < 0.01.

After treatment with risperidone for 2 weeks, compared with baseline, the level of BDNF was significantly increased in ER group (*t* = −2.969, *p* = 0.004), but not in ENR group (*t* = −1.204, *p* = 0.232). Although the level of BDNF in group ER and ENR was still lower than that in HC group, the level of BDNF in group ER was significantly higher than that in group ENR (*t* = 2.632, *p* = 0.01).

### 3.2 Baseline and improvement of PANSS scores comparison between ER patients, ENR patients and HCs

At baseline, the PANSS total score and its subscores (positive subcore and negative subscore) had no statistically significant differences between ER and ENR groups. After 2 weeks treatment with risperidone, PANSS total score was decreased in ER (*t* = 4.623, *p* < 0.001) and ENR (*t* = 2.872, *p* = 0.005) groups, and also had significants between ER and ENR group (*t* = 2.937, *p* = 0.004). There was no significant between ER group and ENR group in drug dose (Table 2).

**TABLE 2:** Positive and negative symptoms scale (PANSS) scores at baseline and after 2 weeks of treatment.

Variable	ER (*n* = 41) mean ± S.D.	ENR (*n* = 48) mean ± S.D.	Analysis 1		Analysis 2		Analysis 3	
			*t*	*p*	*t*	*p*	*t*	*p*
Drug dose (mg) 2 weeks later	3.43 ± 0.43	3.43 ± 0.43	−0.105	0.917				
PANSS total Score baseline	96.78 ± 17.58	98.63 ± 16.48	−0.505	0.615				
P subscore	22.00 ± 4.05	20.75 ± 4.76	1.322	0.190				
N subscore	14.12 ± 3.17	15.58 ± 6.30	−1.346	0.182				
PANSS total Score 2 weeks later	80.40 ± 13.82	89.37 ± 14.38	2.937	0.004**	4.632	<0.001**	2.872	0.005**

*Note*: Analysis 1: ER group versus ENR group; Analysis 2: ER group, PANSS total score baseline versus 2 weeks later; Analysis 3: ENR group, PANSS total score baseline versus 2 weeks later.

Abbreviations: ENR, early non‐response; ER, early response; HC, healthy control; N subscore, negative subscore, P subscore; positive subscore.

**p < 0.01.

### 3.3 Relationship between changes in BDNF levels or baseline BDNF levels and changes in PANSS score after treatment

**FIGURE 1:**
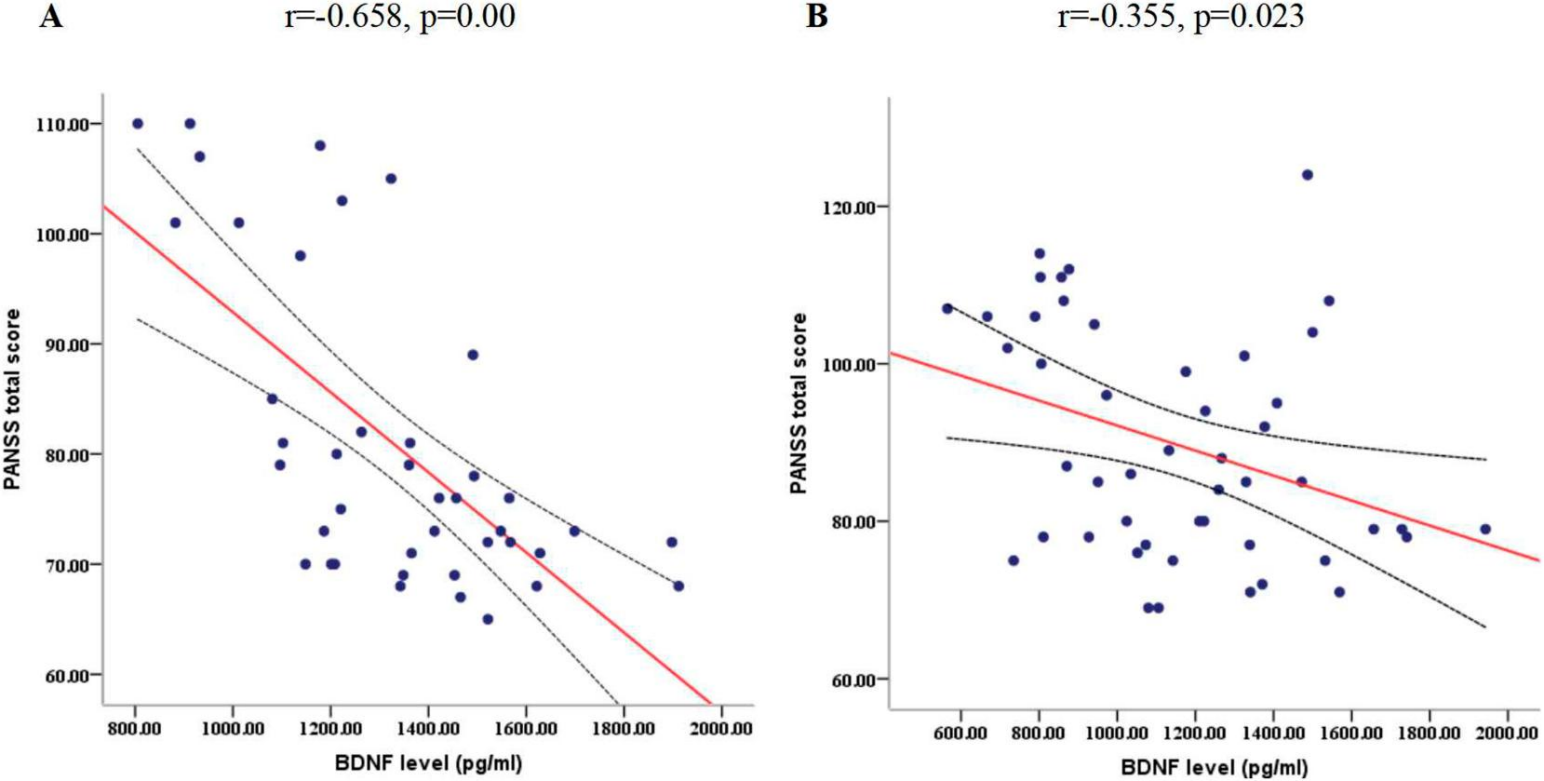
Correlation between changes in brain derived neurotrophic factor (BDNF) levels and changes in positive and negative symptoms scale (PANSS) total score in early response (ER) and early non‐response (ENR) patients after 2 weeks of risperidone monotherapy.

The patients in ER group showed significantly increased serum BDNF levels afte risperidone treatment for 2 weeks, but not in ENR group. Moreover, changes of BDNF levels were negatively associated with changes of PANSS total score (*r* = −0.658, *p* < 0.01 in ER group, *r* = −0.355, *p* < 0.05 in ENR group) (Table 3). Linear regression analysis showed that the change in BDNF levels was an independent predictor for psychotic symptoms improvement in 2 weeks of treatment. (*β* = −0.664, *t* = −5.55, *p* < 0.001 in ER group; *β* = −0.35, *t* = −2.536, *p* = 0.015 in ER group) (Figure 1).

**TABLE 3:** Correlation between positive and negative symptoms scale (PANSS) total score and serum Brain derived neurotrophic factor (BDNF) in schizophrenia patients.

Variable	1	2	3	4	5	6	7	8
1. ER BDNF baseline	1							
2. ENR BDNF baseline	0.065	1						
3. ER BDNF 2 weeks later	0.684**	−0.019	1					
4. ENR BDNF 2 weeks later	−0.004	0.621**	−0.026	1				
5. ER PANSS baseline	−0.853**	−0.052	−0.668**	−0.006	1			
6. ENR PANSS baseline	0.102	−0.65**	0.051	−0.359*	−0.05	1		
7. ER PANSS 2 weeks later	−0.843**	−0.053	−0.658**	−0.007	0.989**	−0.062	1	
8. ENR PANSS 2 weeks later	0.095	−0.641**	0.072	−0.355*	−0.049	0.992**	0.697	1

*Note*: The unit of measurement for BDNF:µg/L.

Abbreviations: BDNF, Brain derived neurotrophic factor; ENR, early non‐response; ER, early response; PANSS, Positive and Negative Syndrome Scale.

**p < 0.01, *p < 0.05.

## 4 DISCUSSION

The major findings in the present study were that the serum levels of BDNF in first‐episode schizophrenic patients were significantly lower than normal controls even after 2 weeks' treatment with risperidone. However, there was significantly difference in serum BDNF levels between the drug‐naive patients and early‐responded medicated patients. To make it clear, the treatment with risperidone for 2 weeks significantly increase the serum levels of BDNF in early responders, while no significantly difference was seen in non‐responders. The logistic regression analysis showed that the difference of serum BDNF levels was the influencing factor for the treatment, and a higher serum BDNF levels was likely associated with better response in Chinese patients.

In our study, we investigated the serum BDNF levels between responsive and nonresponsive patients after 2 weeks of risperidone treatment. We used less than 20% reduction from baseline PANSS to define non‐response and more than 20% to define response. Previous existing data about serum BDNF levels in schizophrenia patients have been controversial. Some studies have demonstrated that serum and plasma BDNF levels remained unchanged after several weeks of risperidone treatment (Hori et al., 2007; Noto et al., 2021). These findings are compatible with our findings that there were no significant differences of serum BDNF levels in non‐responders. Besides, some studies have been in consistent with our finding that decreased serum and plasma levels were observed both in medicated and non‐medicated schizophrenia patients compared to normal controls (Zakharyan & Boyajyan, 2014). Moreover, and interestingly, some show an increase of BDNF levels in the cingulate cortex or hippocampus of schizophrenia (Lech et al., 2021; Reinhart et al., 2015).

In our study, in contrast to the finding of non‐responders, a significantly improvement of serum BDNF levels in early responders was observed after a two‐week of exposure to risperidone. This result is also consistent with findings related to some other psychiatric diseases. Some previous studies reported that plasma BDNF was significantly increased in the responders to drugs with major depressive disorders, and some found that BDNF Val66Met Met allele has been associated with depression (Notaras et al., 2017; Strauss et al., 2005). A study by Lee and Kim suggested that higher plasma BDNF levels in Korean patients might be associated with better response to risperidone treatment (Lee & Kim, 2009). Zhang also showed that BDNF appears to have a strong association with antipsychotic drug effect (Zhang et al., 2013).

There are some possible explanations for the association between serum BDNF levels and response to treatment. A gene‐based association study (Xu et al., 2010) to risperidone. Besides, some found a dose‐response relationship between the number of minor alleles and antipsychotic treatment response. In a word, genetic variation in BDNF plays an important role in the treatment response in patients with schizophrenia.

In previous studies, BDNF, the gene coding for brain‐derived neurotrophic factor, is also known as a protein that is critical for synaptic plasticity and construction in the CNS (Cabelli et al., 1995). Previous experiments showed that if the BDNF gene was selectively deleted in the murine midbrain‐hindbrian regions, the number of tyrosine hydroxylase‐expressing dopaminergic neurons would be reduced (Baquet et al., 2005). Besides, the Val66Met polymorphism (RS6265), a frequently studied SNP, is found to reduce synaptic plasticity by alerting the intracellular trafficking and packaging of proBDNF (Egan et al., 2003; Notaras et al., 2017). The Met allele can lead to a smaller hippocampal volume (Molendijk et al., 2012; Szeszko et al., 2005) which may be responsible for the poor treatment response according to the mechanism that antipsychotic drugs work partially by enhancing dentate gyrus glutamate transmission or through modulating long‐term potentiation in some areas of hippocampus (Tamminga et al., 2010). Moreover, BDNF interacts with multiple neurotransmitters, influences synaptic signaling and also the survival and differentiation of dopaminergic neurons (Feng et al., 1999), and dopamine is a major target for antipsychotic drugs. Guillin demonstrated that BDNF might play an important part in regulating the expression of dopamine D3 receptor (DRD3) in the nucleus accumbens during development and maintenance in adulthood (Guillin et al., 2001). Interestingly, the DRD3 belongs to the dopamine D2‐like receptors (Sokoloff et al., 1990) which modulates dopamine synthesis, release, neuronal activity, and inhibits effects of dopamine D2 receptor (Adermark et al., 2022). As a result, BDNF indirectly controls the dopamine activities by controlling DRD3 expression.

Altogether, our findings suggest that an increased BDNF is associated with a favorable outcome of antipsychotic treatment in schizophrenia patients. Further studies are required to ascertain a higher serum BDNF levels is likely to predict a better response to treatment.

## AUTHOR CONTRIBUTION

Tong Zhao and SuFang Tang: Data curation, Formal analysis, Investigation, Methodology, Visualization, Writing‐original draft, Writing—review & editing. XiaoLei Gao: Results interpretation and integration, Writing—review & editing, Supervision. Juan Li: Results interpretation and integration, Writing—review & editing. Ran Hao: Writing‐original draft, Preparation. GuangBiao Huang and HaiZhi Chen: Writing‐original draft, Preparation, Supervision, Project administration, Funding acquisition.

## CONFLICT OF INTEREST STATEMENT

The authors declare that there are no conflict of interests.

## Data Availability

The data that support the findings of this study are available on request from the corresponding author. The data are not publicly available due to privacy or ethical restrictions.
